# 

**Published:** 1876-10

**Authors:** 


### Contents of October Number.

### ORIGINAL.

ART. I.—Suggestions as to a method of closing recto-vaginal fistula. By Julius F. Miner, M. D. .......... 83
ART. II.—Clinical Notes. By Julius A. Post, M. D. .......... 85

### PROCEEDINGS OF MEDICAL SOCIETIES.

Proceedings of the Buffalo Medical Association, Sept. 5, 1876.—Reduction of dislocated hip of nine months standing.—Osteo-myelitis.—Necrosis of tibia.—Epithelioma of Vulva, .......... 86
Proceedings October 3d, 1876.—Lead Poisoning, .......... 88

### MISCELLANEOUS.

Suit for Mal-practice involving the Surgery of the Hip Joint, .......... 89
Remarks on the Enucleation of Uterine Fibroids, .......... 104
Opium Antidotes Exposed, .......... 109
A case of Phthisis with a Vomica communicating with a gaseous tumor on the Anterior Aspect of the Chest, .......... 111
Correspondence, .......... 116

### EDITORIAL.

Malpractice and Medical Testimony, .......... 117

BOOKS REVIEWED:

Micro-Photographs in Histology. Normal and Pathological. ... 118
Chirurgie Antiseptique, .......... 120
A Manual of Percussion and Auscultation. By Austin Flint, M.D. 120
Inhalation in the Treatment of Disease. By J. Solis Cohen, M.D. 121
A Treatise on Midwifery. By W. S. Playfair, M. D. .......... 121

Books and Pamphlets Received, .......... 122

